# A New Insight on Phased Array Ultrasound Inspection in MIG/MAG Welding

**DOI:** 10.3390/ma15082793

**Published:** 2022-04-11

**Authors:** José Alonso, Santiago Pavón, Juan Vidal, José Perdigones, Isaac Carpena

**Affiliations:** 1 Applied Physics Department, University of Cadiz, CASEM, Avda. Rep. Sah. s/n, 11510 Puerto Real, Cadiz, Spain; 2 Ship Construction Department, University of Cadiz, CASEM, Avda. Rep. Sah. s/n, 11510 Puerto Real, Cadiz, Spain; santiago.pavon@uca.es (S.P.); juan.vidal@uca.es (J.V.); jose.perdigones@uca.es (J.P.); 3 InnerSpec, C. Sanglas, 13, 28890 Loeches, Madrid, Spain; icarpena@innerspec.com

**Keywords:** MIG/MAG welding, ultrasound inspection, phased array, digital images

## Abstract

Weldment inspection is a critical process in the metal industry. It is first conducted visually, then manually and finally using instrumental techniques such as ultrasound. We made one hundred metal inert/active gas (MIG/MAG) weldments on plates of naval steel S275JR+N with no defects, and inducing pores, slag intrusion and cracks. With the objective of the three-dimensional reconstruction of the welding defects, phased array ultrasound inspections were carried out. Error-free weldment probes were used to provide the noise level. The results can be summarized as follows. (i) The top view obtained from the phased array provided no conclusive information about the welding defects. The values of the echo amplitudes were about 70 mV for pores and cracks, and greater than 150 mV for slag intrusion, all of which showed great variability. (ii) The sectional data did not lie at the same depths and they needed to be interpolated. (iii) The interpolated sectional views, or C-scans, allowed the computation of top views at any depth, as well as the three-dimensional reconstruction of the defects. (iv) The use of the simplest tool, consisting of the frequency histogram and its statistical moments, was sufficient to classify the defects. The mean echo amplitudes were 33 mV for pores, 72.16 mV for slag intrusion and 43.19 mV for cracks, with standard deviations of 8.84 mV, 24.64 mV and 12.39 mV, respectively. These findings represent the first step in the automatic classification of welding defects.

## 1. Introduction

Many methods can be used to create a joint between elements but welding is the most reliable, the cheapest and the most efficient for metal pieces, and it can be used for all types of mechanical sets and metallic components [1,2,3]. A weldment is a located joint between metallic or non-metallic components formed through heating them with or without high pressure and with or without additional materials. The heating process can be driven by gas or electricity. Depending on the materials, equipment and gas welding protection used, each type of weldment requires a specific process and a set of operating parameters to ensure the quality of the joint [1,2,3,4].

In the naval industry, the most common type of metallic welding is the MIG/MAG (metal inert gas/metal active gas, up to the protecting welding gas) process, also called GMAW (gas metal arc welding). This welding process involves the use of a metallic arc with a local atmosphere of inert or active protecting gas, using an expendable electrode. The type of gas depends on the material to be welded [1,2,3,4]. The electrode is usually tubular, with metallic dust in, or flux, in a procedure called FCAW (Flux Cored Arc Welding) [4,5]. MIG/MAG welding is not yet able to be automated because the electrode for FCAW cannot be rolled [4,5,6].

A weldment can be perfect or characterized by the presence of imperfections or discontinuities, but a discontinuity is not necessarily a defect. Pores occur due to incomplete fusion and/or incomplete penetration because of a dirty base material, moisture on the joint surface or electrode, insufficient or improper gas shielding or incorrect welding conditions. Other discontinuities can occur due to the use of an incorrect welding technique, incorrect electrode positioning and an incorrect travel speed. Cracks are probably the most dreaded of all discontinuities. In the welding process, they occur at high temperatures during the solidification of the weld metal and they are named hot cracks.

Weldments must be inspected in order to ensure their quality. The inspection can be performed using destructive or nondestructive methods. There are several nondestructive methods:

(i) Visual inspection;
(ii) Basic manual procedures (such as magnetic dust);
(iii) Radiative techniques based on the use of X-rays; and
(iv) Ultrasound techniques (UTs).

The latter can be further divided based on how the ultrasound is generated and/or emitted into the piece to be inspected: TOFD (time of flight diffraction), PA (phased array) and the EMAT (electromagnetic acoustic transducer). TOFD and PA use the same ultrasound generation principle with different numbers of transducers [7]. The PA method is the UT used in this work.

The results of the PA technique can be digital images and/or comma-separated values files. They can be processed in multiple ways with different and advanced techniques, such as mathematical morphology, neural networks and fuzzy logic [8,9,10,11,12,13,14]. PA provides two views of a joint, the top and the sectional views (C-scans). The top view is accepted as an alternative to X-ray inspection. Some researchers have attempted to perform the three-dimensional reconstruction of the welding defects by combining the above advanced techniques with numerical modeling [15,16,17,18,19].

With the objective of the three-dimensional reconstruction of welding defects (pores, slag intrusion and cracks) and their classification by means of basic tools, we made one hundred welding probes using the MIG/MAG FACW method. Inspections were carried out by means of the phased array technique. The TOFD, X-ray and EMAT techniques give only the top-view [19,20,21]. Hence, the only non-destructive technique that provides information about the three-dimensional structure of a defect is PA.

The noise level was determined using the error-free probes and was eliminated. The data processing was based entirely on the geometry of the problem, taking into account the focal law of each beam, obtaining the top and sectional views of the joints. The top view did not provide conclusive information about welding defects. The values of the echo amplitudes were about 70 mV (maximum) for pores and cracks and 150 mV for slag intrusion, but with a high statistical dispersion.

In order to take advantage of the information in the sectional views, we projected them onto a regular grid, allowing the computation of top view at any depth in the joint and the three-dimensional reconstruction of the defects. To our knowledge, this is the first time this kind of processing was been accomplished. Thus, this study offers two contributions to the literature.

The problem of automatic classification is solved using the simple histogram of the data set, which is used for the three-dimensional reconstruction and its statistical moments. The differences among these are important, in terms of their range, shape and frequency distribution. This is also the first time this kind of analysis has been conducted, which is the other important contribution of this work.

## 2. Materials and Methods

### 2.1. Materials and Welding

Two-hundred steel-grade S275JR+N DIN EN 10025 plates, common in shipbuilding, were used to make one hundred controlled testing weldments using the welding facilities of the School of Naval and Ocean Engineering at the University of Cádiz. The dimensions of the plates were 250 mm long, 150 mm wide and 12 mm thick. The mechanical properties and chemical composition of the material are presented in Table 1 and Table 2, respectively.

**Figure 1:**
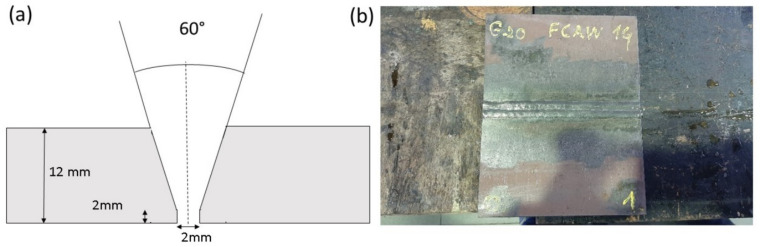
(**a**) Schematic cross-section of the welding probe; (**b**) a finished probe, ready for inspection at the workshop of the School of Naval and Ocean Engineering facilities in the University of Cádiz.

Four groups with the same numbers of probes were considered: 25 with no error (G-probes), 25 with pores (F1-probes), 25 with slag intrusion (F2-probes) and 25 with cracks or fissures (F3-probes). All plates had a 30° chamfer and they were welded using the MIG/MAG FACW process, with a ceramic backing and a final cleaning. All weldments were visually inspected as a first quality test. A schematic cross-section and an image of one of probes are shown in Figure 1.

The material used for the FACW was a FLUXOFIL 14HD of 1.2 mm in diameter. Its mechanical properties and chemical composition are presented in Table 3 and Table 4, respectively.

The welding process was the same as that used in shipbuilding, with seven runs. The technical characteristics are detailed in Table 5. All probes were performed by a professional welder.

The different defects were produced by changing the welding conditions. Pores were obtained by improper gas shielding and slag intrusion by adding slag from previous welds from the same material. Hot cracks were induced by adding some material during runs 2 or 3, such as very short copper or steel wires with a fusion point much lower than the temperature of the torch, and ensuring that the hole was not filled by the new cord.

### 2.2. Phased Array Ultrasound Inspection and Data Processing

The phased array (PA) technique is an advanced ultrasound-based nondestructive technique for weldment inspection, used to detect defects such as pores, slag intrusion and cracks. The combination of beams with multiple angles and different focal depths enables one to carry out a full inspection in just one run.

The array of transducers is sequentially activated in a single housing, together with the temporal sampling windows. The specialized ultrasonic transducer can have from 16 to 256 individual elements, which are pulsed in a programmed pattern. The transducer may be square, rectangular or rounded, and the test frequencies typically range from 1 to 10 MHz.

According to [19], the PA technology has many advantages and some disadvantages. The advantages are: (i) it allows the inspection of complex geometries; (ii) it enables the inspection of metallic and composite materials; (iii) it can be used at high temperatures; (iv) it results in a faster inspection compared with conventional nondestructive techniques as X-rays and (v) it shows an increased detection of flaws compared to other methods. At the same time, PA can be used on composite materials [19], for the inspection of complex steel structures [22] and on complex geometric surfaces with irregular curvature [23]. The technique can also be combined with destructive assays [24]. The main disadvantage is that PA requires permanent physical contact between the transducer and the weldment to be inspected.

In the frame of this work, a Phased Array Sonatest Veo+ system [25] with a 64 pulser/receiver transducer X3A-5M64E-0.6X10 (X3AW-N55S) of high resolution and performance, working at 125MHz, with an A-scan acquisition sampling frequency, was used. There were 55 different focal laws (beams) of 32 elements, covering a range of 55 angles, ranging from 45° to 72°, using a 5 MHz excitation frequency with a 64 element transducer. The parameters were set to ensure the maximum joint coverage with two runs, to the right and to the left of the joint, taking into account the geometry and size of the sensor and the pieces to be welded. The transducer was equipped with a wheel to code the distance along the joint at every millimeter. In addition to the usual calibration used for the ultrasound speed, wedge delay and sensitivity, the option of TCG (Time-Corrected Gain) was also selected to ensure a homogeneous response across the entire area of the sectional view. In other words, the instrumental response to the presence of a defect was the same regardless of its location in the joint.

An inspection was built up on the basis of two runs, to the right and to the left of the joint axis. Both were combined in the data processing stage, taking into account the geometry of the inspection, to obtain a view of the whole joint. The top and sectional views were exported to comma-separated values files. The final data set for each probe consisted of a top view and 250 C-scan sections.

**Figure 2:**
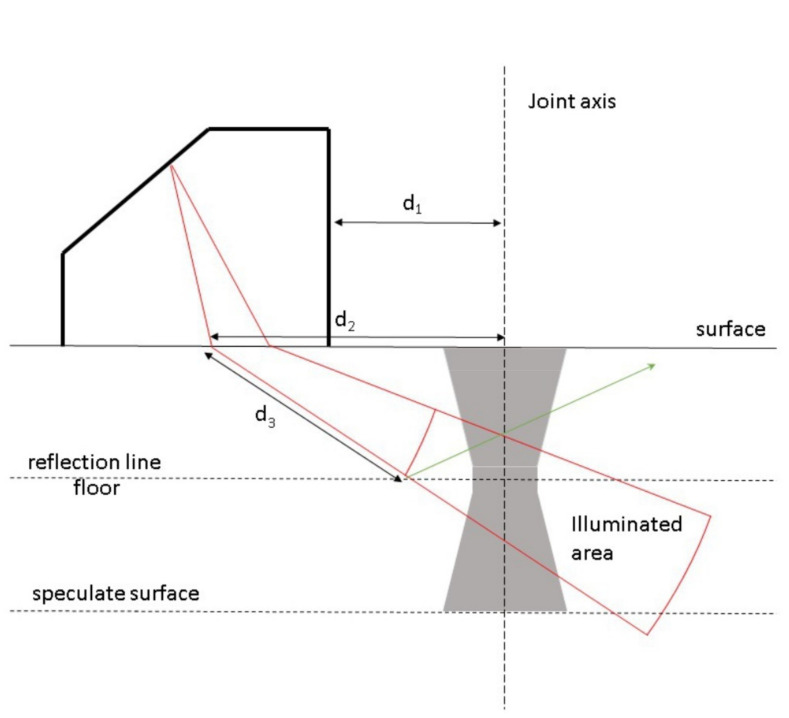
Geometry of the inspection process (see text for explanation).

The geometry of the inspection was as follows (see Figure 2). The origin of the reference frame was located in the axis of the joint. The distance of the transducer to the origin was constant (d1 = 20 mm). The transducer worked with 55 beams, focal laws, with angles ranging from 45° to 72° with an increment of 0.5°. Each beam had a different distance between the incidence point and the axis of the joint (d2). There were 576 measures, starting at a distance d3 from the incidence point with an increment of 0.052 mm on the focal law [25]. It was common to find a representation similar to that shown in Figure 2 with a mirrored image of the weldment because it made it easier to understand the interplay of the incident, refracted and reflected beams. The green ray is an example of a real one.

The raw data were processed using several FORTRAN codes developed by the authors. The comma-separated values files for the top views required little processing, consisting merely of the elimination of the first and last columns in order to remove the border effect. The sectional data are more complex and the geometry of the problem (Figure 2) plays a central role. The code first comprises the accurate computation of the position of each sampled point along each focal law, following the technical specifications of the PA device. Because the data do not lie at the same depths in the weld cord, a kriging interpolation was implemented to bring the data onto a structured regular mesh. The code for the kriging interpolation was taken from [26] and properly adapted. The variograms were estimated before interpolation, and were spherical for all cases. After interpolation, the final data set for each probe consisted of 250 transversal sections with 480 depth levels.

## 3. Results and Discussion

### 3.1. Top View

**Figure 3:**
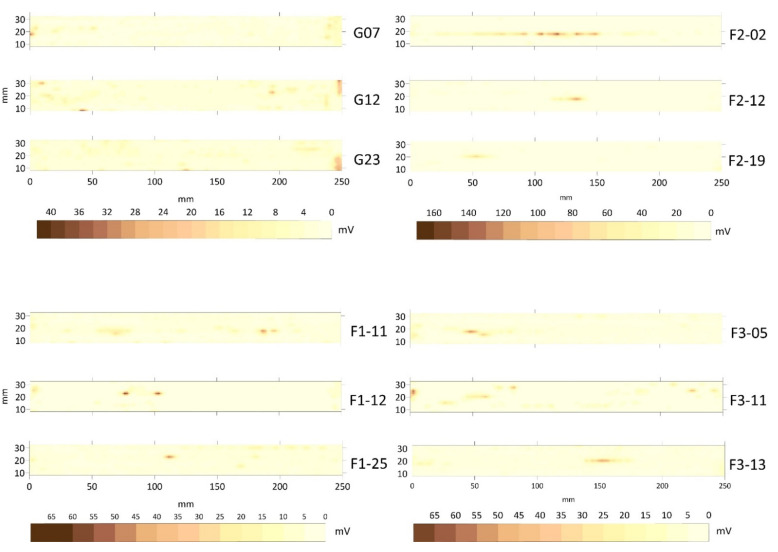
Top view for three cases of probes (G) and the cases of pores (F1), slag intrusion (F2) and cracks (F3). The *x*–axis is the distance from the left side and along the joint in millimeters and the *y*–axis is the transversal distance from the lower limit of the plate. Distances are in millimeters and echoes in millivolts.

The top view is the planar representation of the integrated echoes’ amplitudes inside the joint. It does not provide conclusive information about the size and depth of the defects, only their location along the axis of the joint. The top views for three cases of error-free welding probes (G) and the cases of pores (F1), slag intrusion (F2) and cracks (F3) are presented in Figure 3. The noise level was determined based on the G-probes. The best ones had a mean amplitude value of 3 mV and a variance around 5 mV^2^, and the noise level for the material, welding type and equipment was set to 15 mV, eliminating the first and the last transversal profiles.

As shown in Figure 3, the top views of the F1 probes (pores) had maximum values of amplitude around 40 mV. Pores could be alone (as if F1–12) or concentrated (as in F1–11), but their amplitudes were lower than those of slag intrusion and cracks. F2 probes (slag intrusion) showed very high values of amplitude, around 150 mV, probably due to resonance. Slag intrusion occurred in a chain along the axis of the joint (see F2–02). Finally, the F3 crack probes presented the maximum values, around 70 mV.

It is difficult to identify the type of flaw using only the top views because the signatures of chained pores can be read as cases of slag intrusion and these can be interpreted as a crack. Moreover, it is also not possible to say anything about the depth and the real size of a defect. These observations were pointed out in [20,21], which proposed the combination of two ultrasound-based techniques, TOFD and PA. However, the top view was the only one considered in those studies.

### 3.2. C–Scan or Sectional View

The top view is useful for determining the location of defects along the joint but it does not provide conclusive information about their depth and size in the working piece. This kind of information can be obtained and exploited using the sectional view. After the accurate computation of the position of the sampled data along the focal law of each beam [25] and a kriging interpolation to bring the data onto a regular and structure grid [26], the extraction of the information is straightforward.

**Figure 4:**
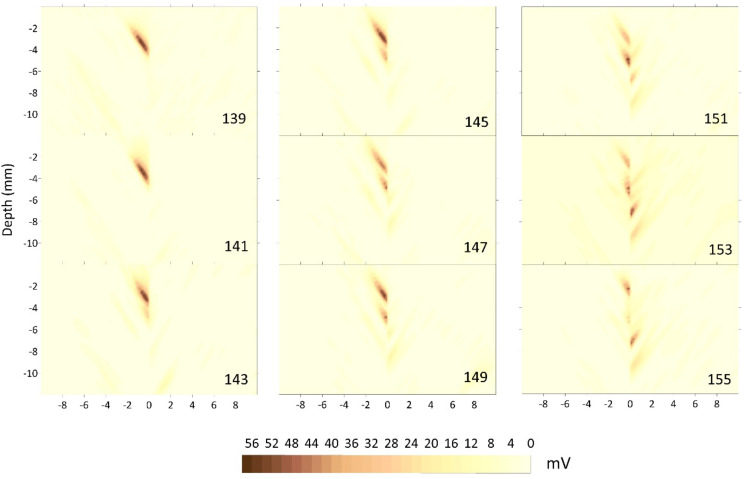
C–scan of the F3–13 probe (crack) from slice 139 to 154, every two millimeters. Echo amplitude is expressed in millivolts and distances in millimeters.

Some kriging-interpolated sectional views of specimen F3–13 are shown in Figure 4. The crack is clearly located at a depth between 2 mm and 4 mm from the surface. The diagonal structure is a result of combining the right and left runs, as in [20,21], of inspections of the joint and it looks similar to the interface of the PA [25].

The information from the top view may be used to select the sectional views to be explored. With the help of the C–scans, it is possible to study the depth of the flaw and its size. Both are greatly important, the first (the top view) for the location and the second (the sectional view) for the vertical size and depth, to study or to inspect a joint.

### 3.3. Top View of Sectional Data

**Figure 5:**
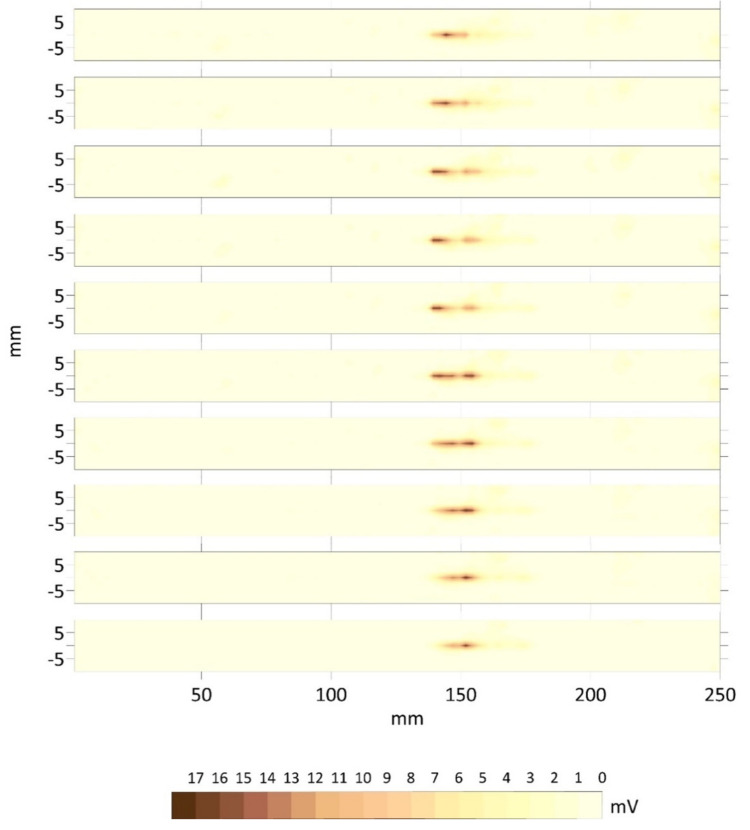
Horizontal top views of the F3–13 probe for the depths of 3.475 mm to 4.35 mm every 0.15 mm.

The sectional data, kriging-interpolated onto a regular grid, can be also used to explore the horizontal distribution of a defect at any depth in the joint. This can be especially important if the horizontal size of the defect must be investigated or for the comparison with other non-destructive inspection techniques providing planar information. Figure 5 shows the slices between 3.475 and 4.350 mm for the F3–13 probe every 0.015 mm. The horizontal structure of the crack in depth indicates the location, as did the top view, and now indicates the extension of the defect. In the case of the analyzed crack, it started as a small defect, grew larger at a greater depth and collapsed.

Some of this information was partially obtained in the analysis of the sectional view because the C–scans allow the determination of the depth and vertical size of the flaw. Here, the top views of the sectional scans provide valuable information about the horizontal size of the flaw, although the determination of the depth can be a quite difficult, depending on the vertical step size at the time of interpolation. The solution for this difficulty is the combination of the top view at depths and sectional scans to obtain a three-dimensional reconstruction of the defect.

### 3.4. Combining Top and Sectional Views: The 3D Reconstruction

**Figure 6:**
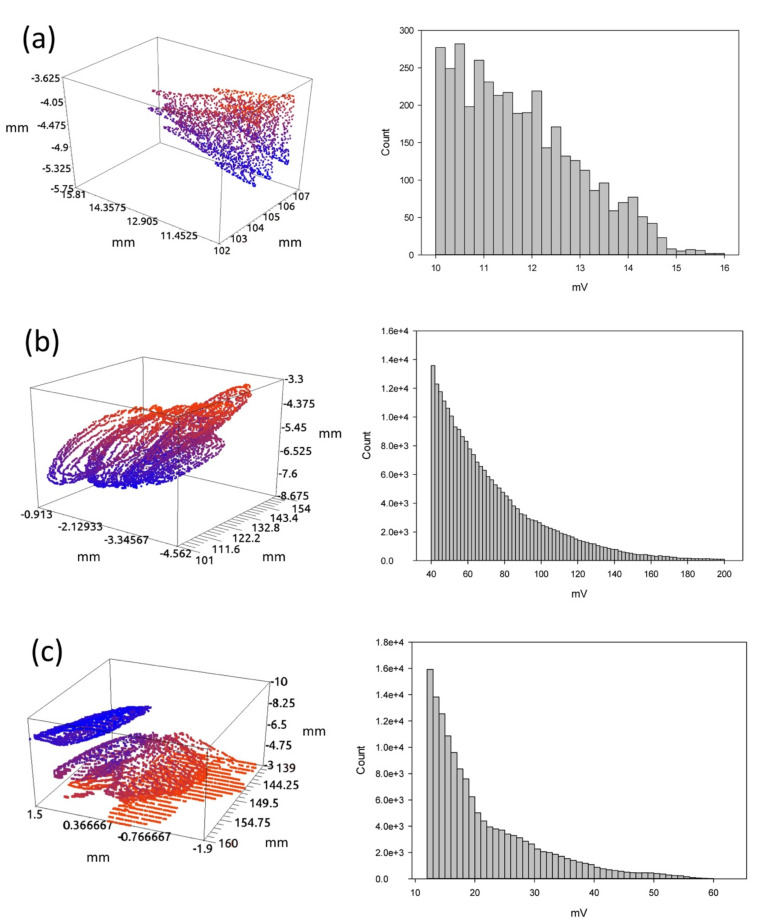
Three-dimensional reconstruction of (**a**) a pore (F1–11), (**b**) slag intrusion (F2–02) and (**c**) crack (F3–13). The three-dimensional reconstructions are on the left and the corresponding histograms are on the right.

It is possible to take advantage of the interpolated vertical sections (C-scans) (Figure 4) and the horizontal top views in depth (Figure 5) to carry out the three-dimensional reconstruction of the defects. Figure 6 shows three reconstructions. Figure 6a shows a pore from F1-21 of which the top view was shown in Figure 3. The three-dimensional view (on the left) shows the shape and size, revealing that the pore has a more complex spatial structure than previously thought. The corresponding histogram is shown on the right, indicating a very small range in the echo amplitude and very low frequency counts. Figure 6b shows the three-dimensional view of a chained slag intrusion in F2–02 at a depth of 4–5.5 mm (on the left). The corresponding histogram is shown on the right. The range can be quite variable, but it is the longest of the three defects. Finally, the crack designated F3-13 is shown in three dimensions in Figure 6c on the left. It affects the working piece in depth. The corresponding histogram is shown on the right. The three-dimensional reconstruction of a welding defect exhibits a short computation time, as it is the first step, together with algorithms for the computation of geometrical properties, to be implemented in an automatic system.

The underlying question is whether there is a simple property or characteristic that is easy to determining and exploit in order to implement the automated classification of defects. The key point is the histogram and its moments.

### 3.5. Classification

The classification of elements and feature extraction in digital image processing is a complex task [27,28]. Some efforts have been made to apply mathematical morphology, neural networks, fuzzy logic, numerical modeling, automatic vision and deep learning to the classification of defects in joints [29,30,31].

Instead of considering advanced techniques for classification, we have looked for the simplest tools available. Based on the results presented in Section 3.4, the clearest differences were present in the histogram of the data set used for the three-dimensional reconstruction of the defect under study. The range, the mean value, the standard deviation and the coefficient of variation for a number of pores, slag intrusion and cracks were computed (Table 6).

As pointed out in Section 3.4, the ranges allow the accurate location of the defect in the bin axis. In addition, the mean values were quite different among the data for pores, around 33.01 mV, whereas we observed values of 72.16 mV for slag intrusion and 43.19 mV for cracks. In the same way, the standard deviation for pores (8.84 mV) was lower than that of slag intrusion (24.64), and the latter was greater that of cracks (12.39 mV).

Based on an inspection of Table 6, there were cases showing amplitude values that were too high or too low. However, this was also the case for the standard deviation for the same defect types due to the unavoidable experimental uncertainty at the time of welding and or inspection. This leads us to think that both values are linked somehow but that they cannot be considered independently. The histogram did not display a bell-shape and the best dispersion measure was relative. The most suitable statistic for expressing the relationship between the mean and the standard deviation is the coefficient of variation. This took values of approximately 6.00 for pores, 2.98 for slag intrusion and 3.48 for cracks. Increasing the number of experimental samples will refine the values.

These results lead us to conclude that the characteristics of the histogram of the data set of pores, slag intrusion and cracks are quite different in their range and in their statistical moments. Hence, they can be used to classify these defects and there is no need to introduce the complexities of advanced techniques such as digital image processing, neural networks, fuzzy logic and numerical modeling.

## 4. Conclusions

Our study of welding defects using PA and their three-dimensional reconstruction led us to the following conclusions:

(i) The top view obtained using the phased array technique provided no conclusive information about welding defects. The values of the echo amplitudes were about 70 mV for pores and cracks, and were greater than 150 mV for slag intrusion, with all of them showing great variability.
(ii) The sectional data did not lie at the same depths and they needed to be interpolated.
(iii) The interpolated C-scans allowed the computation of top views at any depth and the three-dimensional reconstruction of the defects.
(iv) The use of the frequency histogram and its statistical moments was sufficient to classify the defects. The mean echo amplitudes were 33 mV for pores, 72.16 mV for slag intrusion and 43.19 mV for cracks, with standard deviations of 8.84 mV, 24.64 mV and 12.39 mV, respectively.
(v) The range of the histogram and the coefficient of variation were the important statistical properties to consider, as they were quite different for the three studied defects.

## Figures and Tables

**Table 1:** Mechanical properties of the S275JR+N.

Yield Point (ReH MPa)	Tensile Strength (ReN MPa)	Elongation (%)
309	447	31

**Table 2:** Chemical composition (%) of the S275JR+N.

C	Mn	Si	S	P	Cr	Ni	Cu	Al	V	N	CE
0.15	0.84	0.18	0.007	0.016	0.03	0.03	0.06	0.026	<0.005	0.005	0.30

**Table 3:** Mechanical properties of FLUXOFIL 14HD.

Tensile Testing (Rp0.2 MPa)	Tensile Strength (m MPa)	A5 (%)
530	600	25

**Table 4:** Chemical composition (%) of the FLUXOFIL 14HD.

C	Mn	Si	S	P	Cr	Nb	Cu	Al	V	Ti	B
0.049	0.07	0.48	0.008	0.007	0.04	0.01	0.05	0.019	0.03	0.093	0.0035

**Table 5:** Characteristics of the welds: type, current, voltage and gas flux.

Run	Type	I (A)	V (V)	Gas (l/min)
1	Weld root cord	200	24	18
2	Wide weld filler bead	260	27	18
3–4	Thin weld filler bead	260	27	18
5–6–7	Welding combing cord	210	26	18

**Table 6:** Mean (mV), standard value (mV) and coefficient of variation for pores (F1), slag intrusion (F2) and cracks (F3).

F1 (Pores)			F2 (Slag Intrusion)			F3 (Cracks)		
Mean (mV)	Std Dev (mV)	CV	Mean (mV)	Std Dev (mV)	CV	Mean (mV)	Std Dev (mV)	CV
24.70	1.24	19.96	72.65	29.46	2.47	21.43	9.17	2.33
24.51	3.44	7.12	87.68	26.22	3.34	59.57	17.35	3.44
39.98	18.71	2.14	62.51	28.56	2.19	62.05	14.99	4.14
31.94	10.34	3.09	84.31	25.09	3.36	29.37	8.17	3.59
36.96	9.78	3.78	63.52	19.62	3.24	43.52	12.28	3.54
33.40	2.76	12.10	62.32	18.92	3.29			
42.53	15.06	2.82						
30.06	7.78	3.86						
Range: 10–20			Range: 40–200			Range: 15–60		
